# Imaging lexicon for acute pancreatitis: 2012 Atlanta Classification revisited

**DOI:** 10.1093/gastro/gov036

**Published:** 2015-07-27

**Authors:** Binit Sureka, Kalpana Bansal, Yashwant Patidar, Ankur Arora

**Affiliations:** Department of Radiology/Interventional Radiology, Institute of Liver and Biliary Sciences, New Delhi, India

**Keywords:** acute pancreatitis, imaging, acute peripancreatic fluid collection, pseudocyst, walled-off necrosis

## Abstract

The original 1992 Atlanta Classification System for acute pancreatitis was revised in 2012 by the Atlanta Working Group, assisted by various national and international societies, through web-based consensus. This revised classification identifies two phases of acute pancreatitis: early and late. Acute pancreatitis can be either oedematous interstitial pancreatitis or necrotizing pancreatitis. Severity of the disease is categorized into three levels: mild, moderately severe and severe, depending upon organ failure and local/systemic complications. According to the type of pancreatitis, collections are further divided into acute peripancreatic fluid collection, pseudocyst, acute necrotic collection, and walled-off necrosis. Insight into the revised terminology is essential for accurate communication of imaging findings. In this review article, we will summarize the updated nomenclature and illustrate corresponding imaging findings using examples.

## Introduction

Acute pancreatitis is one of the most common gastrointestinal causes of hospitalization. The global incidence of acute pancreatitis varies from region to region and is estimated to be 10–50 cases per 100 000 people per year. The mortality and morbidity due to acute pancreatitis remain unpredictable; however, both are higher in cases of necrotizing pancreatitis. The Atlanta Symposium of 1992 endeavoured to devise a comprehensive framework for grading and categorizing this entity [1]. This classification represented a significant development but, over the subsequent years, it has been found that the classification and its definitions are confusing, leading to inconsistencies in interpretation and reporting [2].

With better understanding of pathophysiology, advances in imaging, and clinical experience with outcomes over the past 20 years, there has been great need for the Association to revise its classification and to provide nomenclature and terminology acceptable to both clinicians and radiologists. In 2012, the Atlanta Working Group and 11 national and international pancreatic societies once again came together and proposed an updated taxonomy for acute pancreatitis that would allow global congruent classification and reporting, and improve patient outcome. The core working group included members from the USA, Netherlands, England, and Greece. With input from pancreatologists of multiple disciplines from around the globe and assistance from an online consensus conference over the internet, they accumulated the evidence-based literature to define and describe the new classification system. The objective of the revised report was to present the amended version of the Atlanta Classification, primarily relating to adults above 18 years of age (Table 1).

**Table 1.** Implications of the revised Atlanta Classification

**The 1992 Atlanta Classification:**
Two types of acute pancreatitis were described: acute oedematous pancreatitis and acute necrotizing pancreatitis. Definitions of pancreatic and peripancreatic collections was confusing and not universal. Various vague terminologies—such as phlegmon, intrapancreatic pseudocyst, organized pancreatic necrosis, necroma, sequestration, pseudocyst associated with necrosis, subacute pancreatic necrosis, and pancreatic abscess—were used.
**The revised 2012 Atlanta Classification:**
Comprehensive classification of definition, types and severity of pancreatitis. New, detailed information on imaging descriptions of various pancreatic and peripancreatic collections. Differentiation between acute peripancreatic fluid, pancreatic pseudocyst, acute necrotic collections and walled-off necrosis. Better communication between radiologists and clinicians. Better patient management and outcomes. Vague terms used previously have been removed. Sterile and infected collections better characterized. Management guidelines of collections defined in a better way. Updated classification of pancreatitis for adults (>18yrs). Consistent, worldwide classification and standardization of terminology. The old 1992 Atlanta classification is obsolete.

### What does the revision add?

The revision takes into account the clinical severity of cases, precisely defines local complications, enables standardized reporting of data, assists in the management of treatment, and facilitates communication between gastroenterologists and radiologists. Most importantly, the revision concentrates on the criteria for diagnosing acute pancreatitis and differentiation between interstitial oedematous and necrotizing pancreatitis, divides the severity of acute pancreatitis into three categories, and defines the various pancreatic and peripancreatic collections. This revision should not be used to formulate strict management protocols, but it does help clinicians formulate basic management guidelines through better classification and stratification of patients according to the severity of their disease [3].

## Definitions

### Definition of acute pancreatitis

Two out of three features are required for diagnosing acute pancreatitis: (i) acute onset upper abdominal pain radiating to the back, (ii) serum lipase or amylase three or more times than the normal range and (iii) classical imaging findings consistent with acute pancreatitis. The actual outset time of acute pancreatitis is the time of onset of symptoms, not the time of entry in the hospital.

Imaging is required when the clinical presentation is classical but the laboratory parameters are inconclusive [4, 5]. Contrast-enhanced CT (CECT) is the modality of choice. Non-contrast and contrast scans (pancreatic phase: 40 seconds after contrast administration) are recommended to accurately assess necrosis and complications. Ultrasound and magnetic resonance imaging (MRI) are adjuncts to computed tomography (CT) and used for the diagnosis of cholelithiasis, choledocholithiasis or disconnected duct syndrome, assessment of the contents of collections, and follow-up imaging. Radiological investigations are usually not required in an emergency setting when the clinical presentation and laboratory markers are consistent with features of acute pancreatitis.

### Types of acute pancreatitis

#### Interstitial oedematous pancreatitis

**Figure 1.**
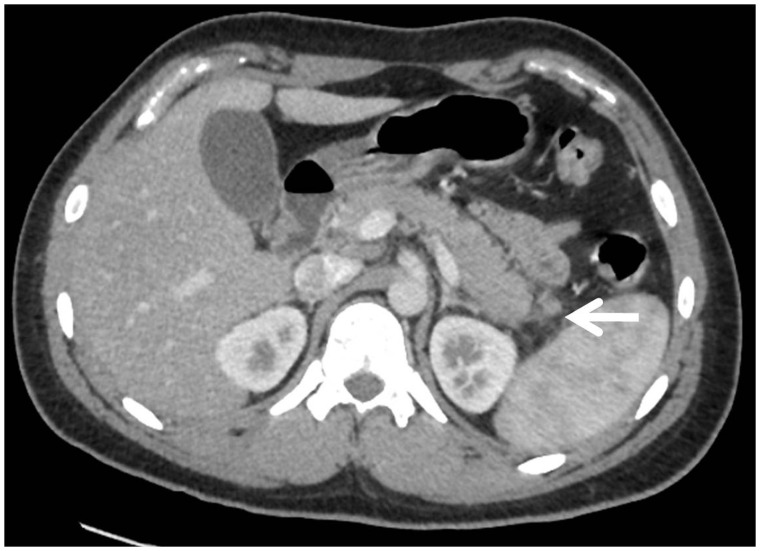
Axial contrast-enhanced CT scan in a 22-year-old male patient with interstitial oedematous pancreatitis, showing mildly increased bulk in pancreatic tail region with peripancreatic fat stranding (arrow).

Eighty to ninety percent of patients with acute pancreatitis have interstitial oedematous pancreatitis, which is usually a milder variant. This type of pancreatitis is characterized by the absence of pancreatic or peripancreatic necrosis on imaging. Homogenous enhancement and diffuse or localized enlargement of the pancreas with/without minimal peripancreatic fluid secondary to inflammatory oedema are visible on CECT. A certain amount of inflammatory haziness or mild stranding exists in the peripancreatic fat (Figure 1). This form of acute pancreatitis usually resolves itself quickly within a week [6].

#### Necrotizing pancreatitis

Five to ten percent of patients with acute pancreatitis develop necrotizing pancreatitis, a severe variant. Necrotizing pancreatitis is classified into three types: (i) combined pancreatic and peripancreatic necrosis (75%), (ii) peripancreatic necrosis alone (20%) and (iii) pancreatic necrosis alone (5%) [7, 8]. The presence of pancreatic parenchymal necrosis characterizes more severe disease than peripancreatic necrosis alone.

Pancreatic perfusion alteration and the necrosis may not be evident in the early disease on imaging. The impairment in pancreatic microcirculation and signs of peripancreatic necrosis evolve over several days, which explains why an early CECT may downgrade the actual levels of necrosis. So on CECT, pancreatic necrosis is more evident around 72 hours after onset of symptoms or after an interval of 5–7 days. On CECT, pancreatic necrosis is seen as an area of impaired or decreased enhancement.

The necrotic area is even better delineated after approximately one week, when the contents have liquefied. The percentage of necrotic parenchyma should be estimated because it has prognostic significance. Peripancreatic necrosis most commonly involves retroperitoneal fat, but is more difficult to detect than parenchymal necrosis. Features of peripancreatic necrosis detectable under CECT include areas of heterogeneous peripancreatic enhancement that contain liquefied low-attenuation and non-liquefied intermediate-attenuation components [9].

#### Infected pancreatic necrosis

Infection in pancreatic and peripancreatic necrosis is unusual during the first week following an episode of necrotizing pancreatitis. Early cases are usually sterile and interstitial oedematous pancreatitis does not become infected. There is speculation that infection occurs when there are air foci in the pancreatic and/or peripancreatic tissues, or the necrotic area is positive for microorganisms by culture and staining. The development of secondary infection in pancreatic necrosis is associated with increased morbidity and mortality. The term ‘pancreatic abscess’ is not used in the revised Classification.

### Phases of acute pancreatitis

In general there are two overlapping phases of acute pancreatitis: the early and the late phases. The early phase lasts for 1–2 weeks, during which the systemic manifestations are linked to the host’s immune response to the local pancreatic injury caused by activation of cytokine cascade. This manifests as systemic inflammatory response syndrome (SIRS) and/or the compensatory anti-inflammatory syndrome (CARS) that can predispose the patient to infection. Organ failure is more likely when SIRS or CARS persists [10–12]. Severity in the early phase depends on whether or not there is organ failure.

The late phase of acute pancreatitis persists beyond two weeks of onset. It is characterized by ongoing systemic inflammation, complications, and/or organ failure. This phase is more commonly seen in moderately severe or severe acute pancreatitis.

### Severity of acute pancreatitis

The severity of acute pancreatitis should be classified for three main reasons: first, to triage the patients and identify those requiring aggressive early treatment; second, for referral of such patients to a specialized care and third, for stratifying these patients into subgroups based on complications and organ failure.

The revised Atlanta Classification divides acute pancreatitis into three grades: mild, moderately severe, and severe acute pancreatitis (Table 3): -

- Mild: lacks organ failure, loco-regional as well as systemic complications. The episode resolves itself within a week, mortality is rare and pancreatic imaging is often not required.
- Moderately severe: distinguished by the presence of transient organ failure, loco-regional as well as systemic complications and/or transient organ failure. The morbidity and mortality are greater than those of mild acute pancreatitis. The event usually does not last more than 2–3 weeks.
- Severe: characterized by persistent organ failure and loco-regional as well as systemic complications. Patients with severe acute pancreatitis that develops within the early phase have a higher mortality rates (36–50%) [10 11, 13]. Development of infected necrosis carries a grave prognosis. On imaging, the morphological severity of pancreatitis is assessed using the CT Severity Index developed by Balthazar *et al.* [14], and the Modified CT Severity Index recently developed by Mortele *et al.* [15] (Table 4).

**Table 2.** Modified Marshall Scoring System for organ dysfunction

Organ system Involved	Score = 0	Score = 1	Score = 2	Score = 3	Score = 4
Respiratory (PaO^2^/FIO^2^)	>400	301–400	201–300	101–200	≤101
Renal (serum creatinine, mg/dL)	<1.4	1.4–1.8	1.9–3.6	3.6–4.9	>4.9
Cardiovascular (systolic blood pressure, mmHg)	>90	<90	<90	<90	<90
		Responsive to fluid resuscitation	Not responsive to fluid resuscitation	pH < 7.3	pH < 7.2

FIO_2_ = fraction of inspired oxygen; PaO_2_ = partial pressure of oxygen in arterial blood

**Table 3.** Severity grading for acute pancreatitis

Severity	Complications		
	Organ failure	Local complications	Systemic complications
Mild	−	−	−
Moderately severe	Transient (<48 hrs)	+	+
Severe	Persistent (>48 hrs); single or multiple organ failure	+	+

**Table 4.** Scoring systems for acute pancreatitis

Pancreatic characteristics	CT severity index (0–10)	Modified CT severity index (0–10)
Pancreatic inflammation		
Normal pancreas	0 (A)	0
Focal/diffuse enlargement of pancreas	1 (B)	2
Peripancreatic inflammation	2 (C)	2
Single acute fluid collection	3 (D)	4
Two or more acute fluid collection	4 (E)	4
Pancreatic necrosis		
None	0	0
Less than 30%	2	2
Between 30% and 50%	4	4
More than 50%	6	4
Extrapancreatic complications^a^	0	2

^a^Including pleural effusion, ascites, vascular, parenchymal and gastrointestinal tract complications

The classification of severity depends on whether organ failure and associated complications are present or absent. Mild acute pancreatitis usually improves within days, while moderately severe acute pancreatitis resolves itself slowly and may require extended in-patient care and intervention. Severe acute pancreatitis demands a longer hospital stay and can also be associated with systemic complications, multi-organ failure and death.

### Definition of organ failure (persistent or transient)

The most reliable marker for disease severity in acute pancreatitis is persistent organ failure (lasting more than 48 hours). The Modified Marshall System (Table 2) is used and it evaluates three organ systems: respiratory, cardiovascular, and renal [16]. A score of 2 or more over a period of more than 48 hours for any one of the three organ systems is defined as persistent organ failure. Transient organ failure is also important in the classification of moderately severe acute pancreatitis and involves a similar scoring system, but is present for less than 48 hours. This scoring system has to be continuously monitored during the disease process and hospital stay, to reassess the level of severity [17].

### Definition of local complications

The original Atlanta Classification differentiated uncomplicated interstitial pancreatitis from acute pancreatitis associated with ‘local complications’. The description of pancreatic and peripancreatic collections was confusing and was not standardized across the globe. The pathophysiology and consequences of collections are now better understood and defined objectively as acute peripancreatic fluid collection (APFC), pancreatic pseudocysts, acute necrotic collection (ANC), and walled-off necrosis (WON).

Local complications should be suspected when there is persistent abdominal pain, increasing levels of serum pancreatic enzymes despite treatment, organ failure and clinical signs of sepsis. Imaging is warranted in these patients, to detect local complications, and it should clearly indicate the location of collections, nature of content within the collection (pure liquid, intermediate, solid and/or gas), wall thickness and extent of pancreatic necrosis. However, the presence or absence of local complications alone does not reveal the severity of the disease process [18, 19]. Other local complications include bowel necrosis, thrombosis of the splenic/portal vein, and gastric emptying malfunction.

### Definition of systemic complications

Renal, circulatory, respiratory organ failures or exacerbation of serious pre-existing illness related directly to acute pancreatitis are examples of systemic complications. This is related to systemic inflammatory response syndrome (SIRS) that accompanies acute pancreatitis for e.g. exacerbation of underlying cardiovascular event, chronic diabetes, obstructive lung disease, chronic liver disease, etc.

### Pancreatic and peripancreatic fluid collections

**Figure 2.**
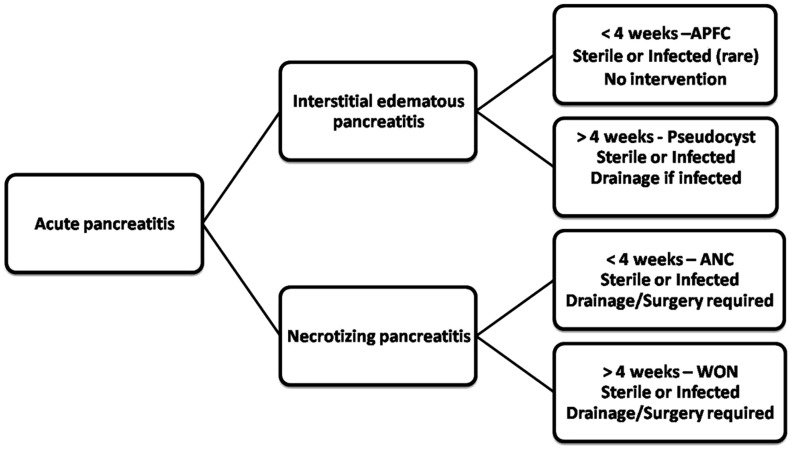
Flow chart showing key lexicons in revised Atlanta pancreatitis nomenclature. APFC = acute pancreatic fluid collection; ANC = acute necrotic collection; WON = walled-off necrosis.

The revised classification defines four types of collections, depending upon the type of acute pancreatitis. Collections associated with acute interstitial oedematous pancreatitis are of two types: APFC and pseudocyst. Collections associated with necrotizing pancreatitis are also divided into two categories: ANC and WON. The collections are divided into two categories in each type, on the basis of the length of time between symptom onset and development of the collection, the presence of a wall surrounding the collection, or the presence of infection (Figure 2).

### Acute peripancreatic fluid collection

**Figure 3.**
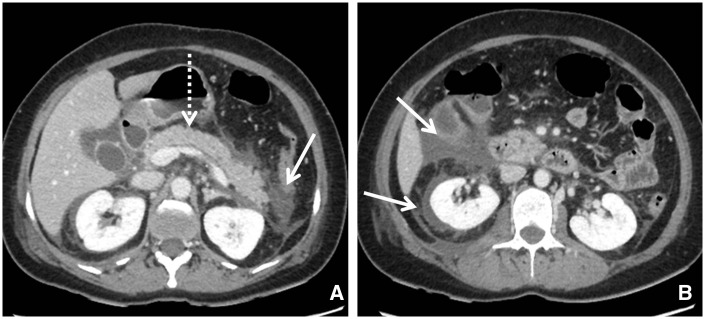
(A and B) Axial contrast-enhanced CT scan in a 53-year-old male with acute interstitial pancreatitis and acute peripancreatic fluid collection (APFC) in the peripancreatic, right anterior pararenal and perirenal spaces (arrows). The pancreas shows homogeneous enhancement (dashed arrow). APFC has fluid density without an encapsulating wall.

On CECT, APFCs do not have a well-defined wall, are homogeneous in appearance, without any solid component, and are restricted by normal anatomical planes of retroperitoneum (Figure 3). APFCs may be single or multiple and usually develop in the early phase of pancreatitis. Most acute fluid collections remain uninfected and usually resolve themselves spontaneously. If the fluid collection persists for more than four weeks, it is likely to develop into a pancreatic pseudocyst [20].

### Pancreatic pseudocyst

**Figure 4.**
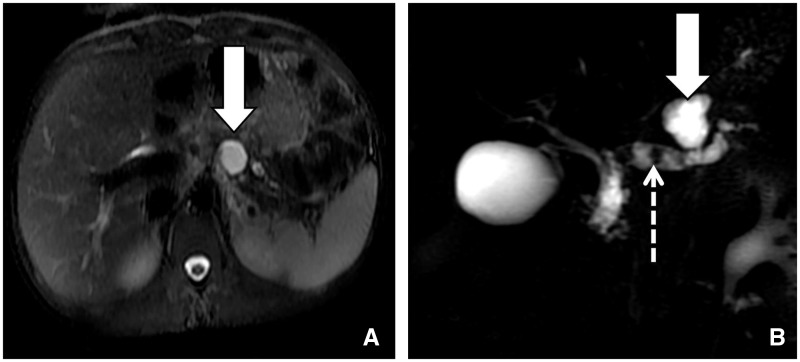
Pseudocyst in a 21-year-old male patient 5 weeks after acute interstitial pancreatitis. (A) Axial T2-weighted MR image, showing encapsulated small T2-hyperintense collection (arrow) with a well-defined wall. (B) Coronal 2D-MR cholangiopancreatography image showing dilated main pancreatic duct with intraductal calculi (dashed arrow) and ductal communication of the pseudocyst.

On CECT or MRI, pseudocyst appears as peripancreatic—or occasionally intrapancreatic—simple collections without any solid component, which have developed perceptible walls within 4 weeks of symptom onset in the setting of APFCs (Figure 4). Increased amylase activity is seen in aspirated fluid. A pancreatic pseudocyst is thought to accrue due to continuous steady leakage of pancreatic juice, usually for more than 4 weeks. This is due to separation of the main pancreatic duct or its side branches in the absence of pancreatic parenchymal necrosis.

#### Can a pseudocyst be seen in necrotizing pancreatitis?

The answer is “yes” when a pseudocyst arises in the setting of acute necrotizing pancreatitis as a result of a ‘disjointed duct syndrome’. Pseudocyst may be evident in necrotizing pancreatitis after surgical necrosectomy. This occurs as a result of localized continuous leakage from the disjointed duct into the operation site, with persistence of necrotic as well as healthy, viable pancreatic tissue [21]. An infected APFC or pseudocyst is uncommon but should be suspected on imaging if gas is observed within the collection.

### Acute necrotic collection

**Figure 5.**
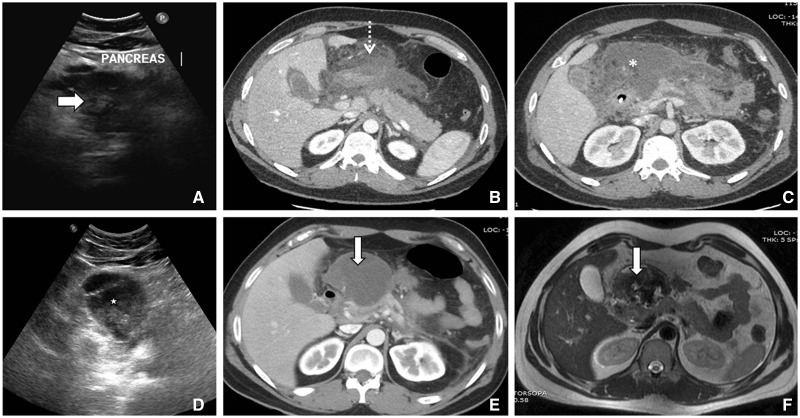
Serial images of a 26-year-old male presenting with acute necrotizing pancreatitis. (A) First ultrasound showing bulky heteroechoic pancreatic (arrow) echotexture. (B) Axial contrast-enhanced CT, showing acute necrotic collection (dashed arrow) with absence of a well-defined wall involving predominantly the peripancreatic region and neck of pancreas. (C) Subsequent axial CT scan around 15 days later, showing increasing heterogeneity (asterix) with areas of ill-defined wall. (D) Ultrasound 4 weeks later than the first ultrasound, showing walled-off necrotic collection (star) in the form of well-defined wall with coarse internal echoes. (E) Axial CT and (F) T2-weighted MR image showing walled-off necrosis (arrow).

Collections associated with necrotizing pancreatitis in the first 4 weeks are called acute necrotic collections. These collections contain variable amounts of solid and inhomogeneous components compared with APFCs, which are simple fluid collections without any soft tissue. On CECT, this type of collection may show loculation, multiplicity and contain a solid component (Figure 5).

Caution must be exercised during the initial 1–2 weeks because, on imaging, it may be difficult to differentiate an APFC from an ANC. At this stage, both types of collection may show fluid density, without any solid component. It is usually after the first week that a distinction between these two important types of collection becomes evident. Collection associated with pancreatic parenchymal necrosis is ANC and not an APFC. Other imaging modalities may help to confirm the presence of a solid component in the collection.

### Walled-off necrosis

**Figure 6.**
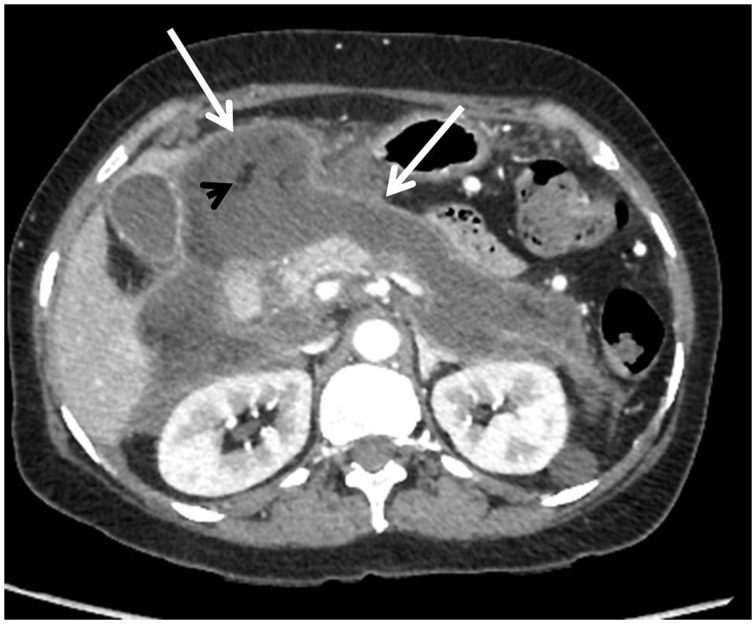
CECT of a 51-year-old female showing walled-off necrosis (WON) after an episode of necrotizing pancreatitis. WON is seen in the pancreatic- as well as peripancreatic region and is characterized by well-defined, thick, enhancing wall (arrows). Collection appears heterogeneous with evidence of liquefied as well as non-liquefied components in the form of areas of trapped fat (arrowhead).

**Figure 7.**
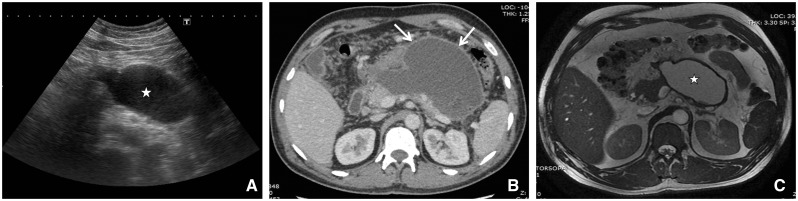
Diagnostic images from a 36-year-old with walled-off necrosis after acute necrotizing pancreatitis. (A) Ultrasound showing collection with well-defined wall and coarse internal echoes (asterix). (B) Axial contrast-enhanced CT showing heterogeneous collection with well-defined wall (arrows). (C) MR axial fast imaging employing steady-state acquisition image (FIESTA) showing WON (asterisk) with thick wall and necrotic debris.

WON matures after four weeks and is well-encapsulated, with a well-defined inflammatory wall (Figures 6 and 7). It is sequelae of ANC, and indicated by the presence of a well-defined, reactive wall. The collection may be infected, may be multiple, and may be present at sites distant from the pancreas. The presence of ductal communication may alter its management.

### Sterile *vs.* infected collections

**Figure 8.**
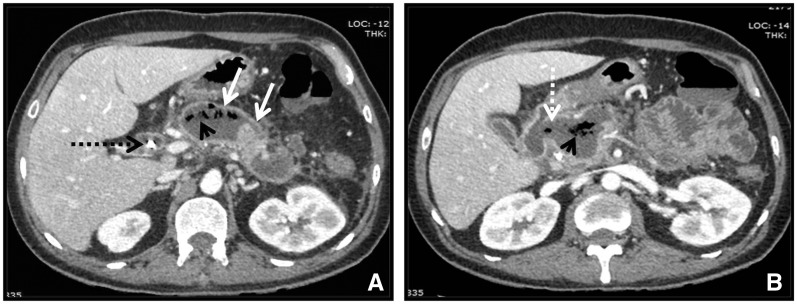
Images from a 44-year-old male 6 weeks after an episode of necrotizing pancreatitis with walled-off necrosis (WON). (A) Axial contrast-enhanced CT showing thick enhancing wall (arrows) around a walled-off necrotic collection with air foci (arrowheads). Also note dilated common bile duct with stent in situ (black dashed arrow). (B) Caudal image showing communication of WON with medial wall of duodenum (white dashed arrow).

All four types of collections mentioned above may be sterile or infected. Infection in a collection is suspected when gas exists within a collection (highly specific), thick-enhancing walls, clinical findings and biochemical profile correspond with signs of infection and/or when needle-guided aspiration confirms the presence of pus. Diffusion-weighted (DW) MRI can be used as a non-invasive technique for the detection of infected collections. Infected collections show peripheral bright signals on DW-MRI images, whereas sterile collections do not show diffusion restriction. Communication between collection and gastrointestinal tract also leads to trapped air foci, which may be mistaken for an infected collection (Figure 8). Infection in APFC and pseudocyst is extremely rare; however it is seen more commonly with ANC and WON. Drainage and surgery is also more often required in collections associated with necrotizing pancreatitis [9, 22, 23].

### Disconnected Pancreatic Duct Syndrome

This anatomic condition is usually seen in necrotizing pancreatitis. It occurs when the pancreatic duct lacks continuity with the viable pancreatic tissue so that this segment no longer drains into the duodenum. On imaging it is diagnosed when there is necrosis of at least 2 cm of pancreas, viable upstream pancreatic tissue, the angle of collection and the pancreatic duct is nearly 90° and active contrast extravasation is seen from the pancreatic duct [24]. Three types of disconnected pancreatic duct syndrome (DPDS) are recognized: concurrent DPDS, delayed DPDS, and chronic pancreatitis-associated DPDS. CECT shows necrosis of the neck and body of the pancreas, but perfusion of the tail in concurrent DPDS. Delayed DPDS is recognized on imaging as a pseudocyst or WON occupying the mid-gland with perfused left-sided pancreatic remnant. Chronic pancreatitis-associated DPDS is associated with a stricture or stone in the proximal duct, leading to atrophy of the duct and, therefore, a disconnected duct results in pseudocyst formation. DPDS may be associated with pancreatic fistulas, which are classified as (i) internal if the pancreatic duct communicates with the peritoneal or pleural cavity or another hollow viscus and (ii) external if the pancreatic duct communicates with the skin [25].

## Conclusion

The revised Atlanta Classification of acute pancreatitis has been able to provide a kaleidoscopic view of the dynamics involved in the evolution of acute pancreatitis. Various renowned national and international societies—such as the American Pancreatic Society, International Association of Pancreatology, European Pancreatic Club, American Gastroenterological Association, Society for Surgery of the Alimentary Tract, the Pancreas Club—have endorsed the new classification.

The terms previously in use—such as phlegmon, intrapancreatic pseudocyst, organized pancreatic necrosis, necroma, pancreatic sequestration, pseudocyst associated with necrosis, subacute pancreatic necrosis, and pancreatic abscess—are no longer used. The accurate description of two phases (early and late), two types (interstitial oedematous pancreatitis and necrotizing pancreatitis), disease severity (mild, moderately severe, and severe), and local fluid and solid pancreatic and peripancreatic collections based on the characteristics of fluid and necrosis, have improved communication between radiologists and gastroenterologists, as well as patient stratification, easing and ensuring consistency in the investigation and reporting of data in research. The new terms are summarized in Table 5.

**Table 5.** Revised nomenclature of acute pancreatitis, based on CECT

**Interstitial oedematous pancreatitis:**
Acute inflammation of the pancreatic parenchyma and peripancreatic tissues without necrosis
CECT criteria:
•Homogeneous pancreatic parenchyma enhancement •No evidence of necrosis •May be associated with APFC or pseudocyst
**Necrotizing pancreatitis:**
Inflammation associated with pancreatic and/or peripancreatic necrosis
CECT criteria:
•Areas of non-enhancing pancreatic parenchyma •Presence of peripancreatic necrosis (ANC or WON)
**Acute peripancreatic fluid collection (APFC):**
Peripancreatic fluid associated with interstitial oedematous pancreatitis in first 4 weeks
CECT criteria:
•Homogeneous collection of fluid density •No definable wall
**Pancreatic pseudocyst:**
Peripancreatic fluid collection associated with *interstitial oedematous pancreatitis* after 4 weeks
CECT criteria:
•Homogeneous fluid density collection •Presence of well-defined wall •No solid component
**Acute necrotic collection (ANC)**
Peripancreatic collection associated with necrotizing pancreatitis in first 4 weeks
CECT criteria:
•Heterogeneous collection with non-liquid density •No perceptible wall •May be intrapancreatic and/or extrapancreatic
**Walled-off necrosis (WON):**
Peripancreatic collection associated with *necrotizing pancreatitis* after 4 weeks
CECT criteria:
•Heterogeneous collection with liquid and non-liquid density •Presence of well-defined wall

*Conflict of interest statement:* none declared.
